# Machine Learning Applications in Medical Image Analysis

**DOI:** 10.1155/2017/2361061

**Published:** 2017-04-13

**Authors:** Ayman El-Baz, Georgy Gimel'farb, Kenji Suzuki

**Affiliations:** ^1^Bioengineering Department, The University of Louisville, Louisville, KY, USA; ^2^Department of Computer Science, The University of Auckland, Auckland, New Zealand; ^3^Department of Radiology, The University of Chicago, Chicago, IL, USA

Multiple today's medical imaging modalities, for example, X-ray CT, MRI/fMRI, and PET scanners, supply computer-aided diagnostics (CAD) with a host of complex and highly informative images. The resulting big volumes of raw visual information are extremely difficult to handle. Thus new strategies for imaging-based CAD and therapies of diseases have to be developed.

In recent years, machine learning became one of the major tools of medical image analysis in various CAD applications. Prior knowledge being learnt from characteristic examples provided by medical experts helps to guide image registration, fusion, segmentation, and other computations towards accurate descriptions of the initial data and extraction of reliable diagnostic cues to reach the CAD goals. Inspired by and combined artificial intelligence, pattern recognition, biology, mathematical statistics, optimization, and many other fields of science, machine learning is successfully employed to find hidden relationships in the complex image data and link them to the goal diagnoses or monitoring of diseases. For a very simple example, learning quantitative 3D shape descriptors of the* corpus callosum* on brain MRI helps much in organizing a successful early CAD of autism or dyslexia.

This special issue pursues the goals of discussing challenges, technologies, and applications of machine learning in the present CAD. Careful reviewing of more than 31 submissions resulted in the selection of 12 papers covering the following topics: measuring topological DWI tractography to detect Alzheimer's disease; 3D kidneysegmentation from abdominal images; driver fatigue detection based on a single EEG channel; accuracy assessment for iterative closest point (ICP) registration; texture and morphological analyses of multiple regions of interest (ROI) to classify breast ultrasound (BUS) images; pulmonary nodule classification with deep convolutional neural networks; combined lung nodule classification with local difference patterns; automatic lung segmentation from thoracic CT; instrument detection and pose estimation in retinal microsurgery; deep and transfer learning for colonic polyp classification; research on techniques of multifeatures extraction for tongue image and its application in retrieval; and active learning to classify diabetic retinopathy.

N. Amoroso et al. used multiplex network concepts to characterize the brain organization from a topological perspective.

F. Khalifa et al. integrated discriminative features from current and prior visual appearance models into a random forest classifier to automatically segment 3D kidneys from dynamic CT images.

J. Hu combined four entropy features and ten classifiers to detect driver fatigue by processing an EEG.

G. Krell et al. compared different unconstrained ICP algorithms on realistic noisy data from an optical sensor of the tomotherapy HD system.

M. I. Daoud et al. combined multiple-ROI morphological and texture analyses to effectively segment BUS images.

W. Li et al. designed deep convolutional neural networks (CNNs) with strong autolearning and generalization abilities to classify lung nodules.

K. Mao and Z. Deng proposed local difference patterns (LDP) and combined classifiers to specify lung nodules on low-dose CT images.

J. Wang and H. Guo presented a fully automatic three-stage lung segmentation by skin boundary detection, rough determination of a lung contour, and pulmonary parenchyma refinement.

M. Alsheakhali et al. modeled detection, tracking, and pose estimation of a retinal microsurgical instrument as a conditional random field (CRF) inference in order to localize the instrument's forceps tips and center point and estimate the orientation of its shaft.

E. Ribeiro et al. explored automated classification of colonic polyps by deep learning of different pretrained or built from scratch trainable CNNs on 8-HD-endoscopic databases acquired by various imaging modalities.

L. Chen et al. presented a novel approach to extract color and texture features of tongue images. Results showed that the developed approach can improve the detection rate of lesion in tongue image relative to single feature retrieval.

Y. Zhang and M. An used an active learning based classifier of features extracted by recognizing anatomical parts and detecting lesions to identify retinal images and further reduce costs of screening the diabetic retinopathy.

